# Prognostic drivers beyond anatomy: towards tailored surgical management in descending necrotizing mediastinitis

**DOI:** 10.1007/s13304-026-02592-z

**Published:** 2026-03-06

**Authors:** Lubiao Liang, Lin Yuan, Yang Tang, Anping Chen, Cheng Chen, Yongxiang Song, Gang Xu

**Affiliations:** 1 Department of Thoracic Surgery, The Affiliated Hospital of Zunyi Medical University, 149 Dalian Road, Zunyi, 563000 Guizhou China; 2 Department of Thoracic Surgery, The Second Affiliated Hospital of Zunyi Medical University, Zunyi, 563000 Guizhou China

**Keywords:** Descending necrotizing mediastinitis, Prognosis, Risk factors, Surgical procedures, Hypoalbuminemia

## Abstract

The management of descending necrotizing mediastinitis (DNM) is challenging due to diagnostic delays and a lack of consensus on optimal therapeutic. While the Endo classification describes anatomical extent, its link to patient outcomes remains unclear. This study aimed to evaluate the prognostic utility of the Endo classification and identify key factors predictive 90-day mortality and prolonged hospitalization. We performed a retrospective, single-center cohort study spanning 2015–2025 that included 52 consecutive patients with descending necrotizing mediastinitis who were managed surgically. Demographic characteristics, clinical features, Endo classification, operative approaches, and postoperative outcomes were systematically evaluated. Univariable regression and correlation analyses were applied to determine factors associated with 90-day mortality and length of postoperative hospital stay. The cohort (median age 54 years, 65.4% male) had an 11.5% 90-day mortality rate. The Endo classification guided surgical approach but was not significantly associated with mortality or hospital stay. Instead, preoperative hypoalbuminemia (odds ratio (OR) 1.29 per 1 g/L decrease, *p* = 0.032), postoperative white blood cell count (OR 1.40 per × 10^9^ decrease, *p* = 0.046) and odontogenic of infection (OR 0.122, *p* = 0.026) were significant risk factors for mortality. A shorter symptom-to-diagnosis interval (r = − 0.386, *p* = 0.008) and the presence of empyema (median stay 34.5 vs. 25.5 days, *p* = 0.020) were independently associated with a longer postoperative hospital stay. In lower mediastinitis (Types IIA-C), a thoracic approach was associated with a lower incidence of the composite outcome (reoperation/death) compared to a transcervical approach (9.1% vs. 33.3%), though this difference was not statistically significant (*p* = 0.227). In DNM, physiological reserve and disease tempo are more powerful prognostic indicators than anatomical extent alone. A fulminant presentation and nutritional status are key prognostic indicators. These findings support a management paradigm that integrates early physiological optimization with tailored surgical intervention and postoperative care based on disease extent.

## Introduction

Descending necrotizing mediastinitis (DNM) is a rare but highly lethal infection originating from odontogenic or cervicofacial sources, which propagate along the deep fascial planes into the mediastinum. Despite its low incidence, DNM persists as a formidable thoracic surgical emergency, with contemporary mortality rates reported between 3.6 and 25.8% despite advances in critical care and diagnostic imaging [1–3]. This persistently high mortality is largely attributable to diagnostic delays and the lack of consensus on optimal management strategies, particularly regarding the extent of surgical intervention [4].

The seminal work of Estrera et al., later refined by Wheatley et al., established robust diagnostic criteria for DNM, providing a critical foundation for consistent case identification [5]. To further refine the anatomical description of the disease, Endo and colleagues introduced a computed tomography (CT)-based classification system that stratifies DNM based on the caudal extent of mediastinal involvement [6]. This schema, subsequently validated and supplemented by Sugio et al. [7], has become an invaluable tool for conceptualizing the disease’s spread. However, a significant translational gap remains: while the Endo classification describes where the infection is, it provides limited evidence-based guidance on how to best address it surgically.

The core controversy in DNM management revolves around the choice of surgical approach for mediastinal drainage. Options range from the less invasive transcervical approach [8] to various thoracic procedures [9, 10], including video-assisted thoracic surgery (VATS) and mediastinotomy [11, 12]. Current practice is often guided by surgeon preference and institutional protocols rather than high-level evidence, owing to the rarity of the condition and the resultant paucity of comparative studies. Furthermore, the prognostic factors that determine survival and recovery in DNM are not fully elucidated. While the disease’s severity is undeniable, the specific patient-specific and treatment-specific variables that predict a protracted hospital course or mortality remain poorly defined, hindering risk stratification and personalized management.

Therefore, to address these critical knowledge gaps, we conducted a single-center, retrospective cohort study of surgically managed DNM patients over a decade. The aims of this study were to evaluate the correlation between the Endo classification and surgical outcomes, and identify key patient characteristics and clinical parameters predictive of prognosis, with the ultimate goal of informing a more evidence-based and personalized management algorithm.

## Patients and methods

### Study design and patient selection

This single-center, retrospective observational study enrolled all inpatients diagnosed with DNM at the Affiliated Hospital of Zunyi Medical University between May 2015 and May 2025. The diagnosis was confirmed using the criteria established by Estrera and later refined by Wheatley: (1) clinical manifestations of severe systemic infection; (2) characteristic radiographic evidence of mediastinal involvement; (3) intraoperative or histopathological confirmation of necrotizing mediastinal infection; and (4) establishment of a causal linkage to a primary oropharyngeal or cervical infectious source [5]. Patients with mediastinal infections of iatrogenic origin (e.g., post-esophageal intervention or cardiothoracic surgery) were excluded. This study was approved by the Institutional Ethics Committee of the Affiliated Hospital of Zunyi Medical University (No.KLL-2025-568). The requirement for informed consent was waived by the committee due to the retrospective nature of the study. The study protocol adhered to the principles of the Declaration of Helsinki and relevant Chinese data protection regulations.

### Data collection

Demographics and therapeutic data were retrieved from electronic medical records using a pre-defined case report form. Collected variables encompassed:

**Demographics and comorbidities:** Age, sex, body mass index (BMI), and pre-existing comorbidities; **Presentation and diagnosis:** Etiology and primary infection site, symptom-to-diagnosis interval (defined as the duration from the patient’s first reported symptom to the time of definitive hospital diagnosis of DNM), diagnostic imaging modalities, presenting signs/symptoms, and the extent of mediastinal involvement classified according to the Endo system. Imaging characteristics such as empyema, pulmonary infiltrates, or cavitary lesions were recorded. Vital signs and key laboratory values at admission—including white blood cell count, neutrophil percentage, hemoglobin, and albumin—were also collected; **Treatment details:** Total operative time, surgical approaches for mediastinal drainage, number and location of drains, performance of tracheostomy, and results of microbiological cultures; **Postoperative course:** Duration of mechanical ventilation and drain placement, requirement for reoperation, antibiotic therapy, and serial laboratory parameters—including white blood cell count, hemoglobin, and albumin—measured within the first 24 h following surgery; **Outcomes:** Postoperative hospital stay, 90-day in-hospital mortality, date and cause of death (if applicable), and incidence of reoperation. Given the relatively modest sample size and the clinical severity of DNM, the composite endpoint of reoperation or death was used to increase the statistical power to detect a clinically meaningful difference between surgical approaches. Both components represent critical, unequivocal indicators of treatment failure in this context.

### Endo classification

The anatomical extent of DNM was classified based on the system proposed by Endo et al. and supplemented by Sugio et al. [6, 7], as follows: **Type I:** Infection confined to the upper mediastinum, above the level of the carina; **Type IIA:** Infection extending to the lower anterior mediastinum; **Type IIB:** Infection involving both the anterior and posterior lower mediastinum; **Type IIC:** Infection extending to the posterior lower mediastinum.

### Treatment and surgical procedures

All patients underwent a standardized management protocol, including immediate broad-spectrum antibiotics, intensive care support, and urgent surgical intervention. The initial step involved incision and drainage of the primary cervical abscess by oropharyngeal surgeons, including drainage of the primary site, blunt dissection through cervical fascial planes to establish pathways, and packing with gauze.

The approach for mediastinal drainage was determined by the caudal extent of infection on CT (Endo classification): Transcervical Approach: Indicated for infections confined to or extending to the posterior upper mediastinum. A cervical incision was used to digitally or under videoscopic assistance dissect along the retrosternal and peritracheal spaces down to the carina, opening abscess cavities with an electrocautery hook. Thoracic Approach: Employed for infections involving the anterior or entire lower mediastinum. This included video-assisted thoracic surgery (VATS), thoracotomy, or a subxiphoid approach to widely unroof all CT-identified abscess cavities.

### Surgical principle and drainage strategy

The cornerstone of both approaches was radical debridement, defined as the removal of all macroscopically necrotic tissue until viable, bleeding tissue margins were encountered. Following debridement and thorough irrigation (hydrogen peroxide, povidone-iodine, and saline), a structured drainage strategy was implemented:

Transcervical Approach: Typically, two 18F soft silicone drains were placed: one along the pretracheal space and another along the retrosternal space toward the infection focus, ensuring upward drainage. Thoracic Approach: Two large-bore chest tubes (28-32F) were placed for pleural drainage. Additionally, an 18F soft silicone drain was positioned in the most dependent part of the unroofed mediastinal abscess cavity (e.g., posterior to the pericardium) to establish direct, dependent drainage.

### Postoperative management

All patients were managed in the ICU. Drains were maintained until clinical and septic parameters improved, drainage became serous and clear, with an output of less than 10 mL per day. Drains were irrigated with normal saline to maintain patency (no negative pressure suction). Antibiotic therapy was de-escalated based on intraoperative culture results. An early tracheostomy was performed if prolonged mechanical ventilation was anticipated.

### Statistical analysis

The normality of continuous variables was assessed using the Shapiro–Wilk test. Non-normally distributed data are reported as median with interquartile range (IQR) and were compared using the Mann–Whitney U test (for two groups) or the Kruskal–Wallis H test (for multiple groups), with Dunn’s procedure for post-hoc pairwise comparisons where applicable. Categorical variables are expressed as number (percentage) and were compared using Fisher’s exact test. Univariable logistic regression analyses were performed to determine factors associated with 90-day mortality, reporting odds ratio (OR) with corresponding 95% confidence interval (CI). Associations between continuous variables and hospital stay length were examined using Spearman’s rank correlation coefficient. A two-sided p-value of < 0.05 was considered statistically significant. All analyses were performed using the Statistical Package for the Social Sciences (SPSS Version 23.0, IBM Corp., Armonk, NY, USA) or GraphPad Prism (Version 10.2.3, GraphPad Software, San Diego, CA, USA).

## Results

### Patient characteristics

**Fig. 1:**
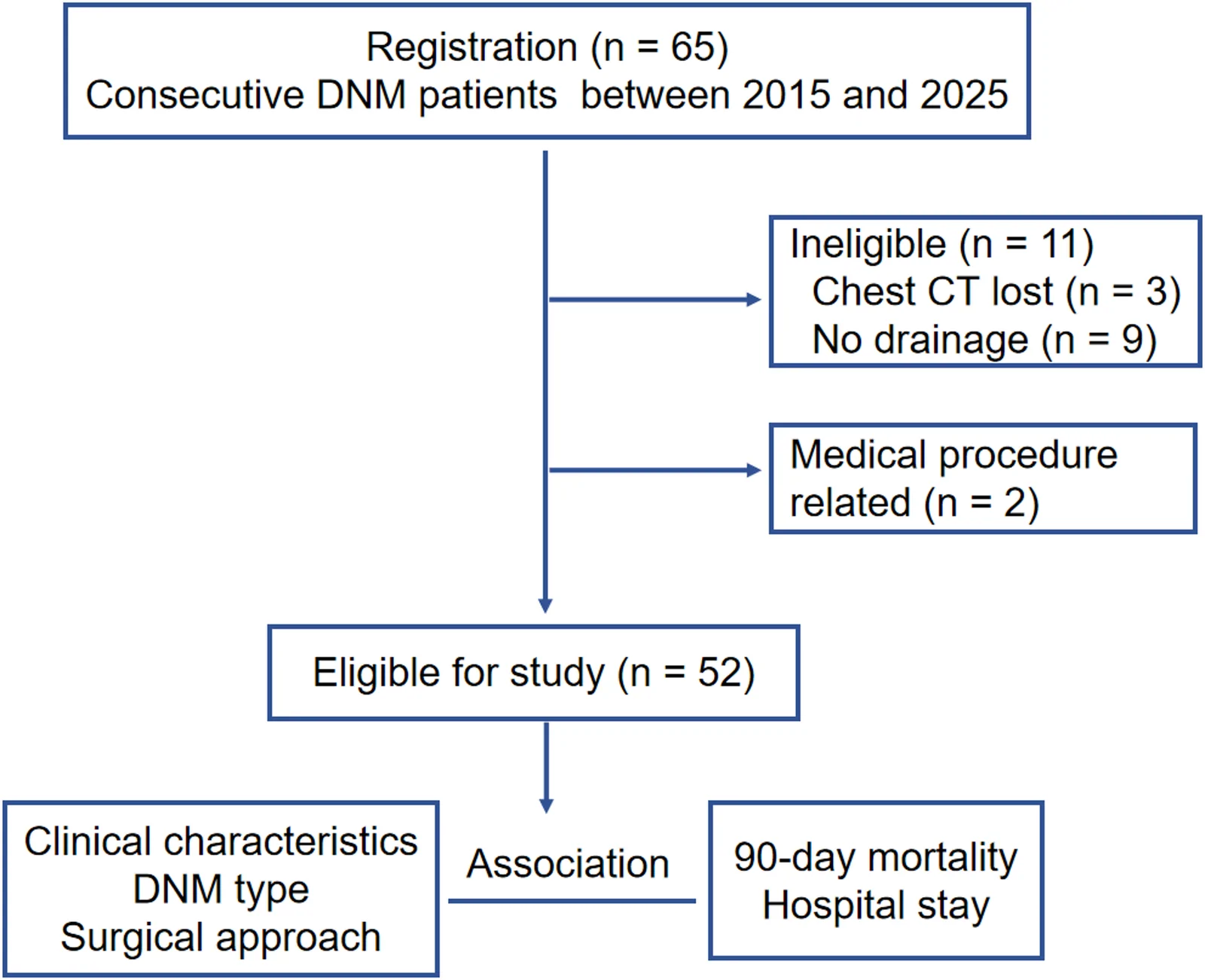
A total of 65 patients were registered and the data of a total of 52 patients were finally eligible for the study. *DNM* Descending necrotizing mediastinitis

During the study period, 65 patients were initially diagnosed with DNM. After applying exclusion criteria—9 patients managed non-surgically, 2 with incomplete imaging data, and 2 with medical related procedures—a final cohort of 52 surgically managed patients was included for analysis (Fig. 1).

Baseline and clinical characteristics are summarized in Table 1. The cohort had a median age of 54 years (range: 19–89) and was predominantly male (65.4%). Most patients (65.4%) were non-smokers. Diabetes mellitus was the most prevalent comorbidity (36.5%), followed by hypertension (19.2%).

**Table 1:** Baseline clinical, laboratory, and microbiological characteristics of the study corhort (n = 52)

Characteristics	Value
Demographics	
Male, n (%)	34 (65.4)
Age (years), Median (range)	54 (19–89)
Smoker, n (%)	18 (34.6)
Comorbidities, n (%)	
Diabetes mellitus	19 (36.5)
Hypertension	10 (19.2)
Malignant neoplasm	5 (9.6)
Others	12 (23.1)
Presenting symptoms, n (%)	
Pain	52 (100)
Fever	36 (69.2)
Neck swelling	47 (90.4)
Dysphagia	50 (96.2)
Others	29 (55.8)
Source of infection, n (%)	
Odontogenic	8 (15.4)
Pharyngeal	31 (59.6)
Unclear or others	13 (25)
Preoperative laboratory data, Median (IQR)	
White blood cells (× 10^9^/L)	15.3 (10.3–20.7)
Neutrophils (%)	89.0 (87.0–91.7)
Hemoglobin (g/L)	132 (125–145)
Serum Albumin (g/L)	29.4 (25.6–35.6)
Pathogen type (n = 45), n (%)	
Gram-Positive Cocci	36 (80.0)
Gram-Negative Bacilli	29 (64.4)
Fungi	4 (8.9)
Identified pathogens (n = 52), n (%)	
*Streptococcus constellatus*	17 (32.7)
*Acinetobacter baumannii*	12 (23.1)
*Streptococcus anginosus*	6 (11.5)
*Klebsiella pneumoniae*	5 (9.6)
Others	29 (55.8)

Pain was a universal presenting symptom (100%). Dysphagia (96.2%) and neck swelling (90.4%) were also highly frequent, while fever was documented in 69.2% of patients.

Initial laboratory investigations revealed a profound systemic infection and nutritional compromise. The median white blood cell count (WBC) was markedly elevated at 15.3 × 10^9^/L (IQR 10.3–20.7), with predominant neutrophilia (median 89%, IQR 87.0–91.7). The median serum albumin level was 29.4 g/L (IQR 25.6–35.6), indicating significant hypoalbuminemia.

### Etiology and microbiological profile

A definitive primary infection source was identified in 39 patients (75.0%). The remaining 13 patients (25.0%) presented without localized symptoms but with systemic manifestations such as fever, neck swelling, pain, and dysphagia. Pharyngeal infections (59.6%) were the most common etiology, followed by odontogenic sources (15.4%).

Pathogen cultures were positive in 45 patients (86.5%). Monomicrobial and polymicrobial infections were identified in 26 (50.0%) and 19 (36.5%) patients. Gram-positive cocci were the predominant isolates (80.0%), followed by Gram-negative bacilli (64.4%) and fungi (8.9%). The most frequently species were *Streptococcus constellatus* (32.7%), *Acinetobacter baumannii* (23.1%), *Streptococcus anginosus* (11.5%), and *Klebsiella pneumoniae* (9.6%).

### DNM staging and surgical management

DNM staging was based on preoperative computed tomography (CT). Associated CT findings are detailed in Table 2. Empyema (intraoperatively confirmed) was observed in 20 patients (38.5%), with a significantly higher incidence in Type IIB (60%) than in Type I (5.3%; *p* = 0.003). Pulmonary infiltrates (51.9%) were significantly more common in Type IIA, IIB, and IIC than in Type I (*p* = 0.028). Cavitary lesions within abscess areas were also commonly noted.

**Table 2:** Imaging features and surgical management by DNM classification

Variable	Total (n = 52)	I (n = 19)	IIA (n = 16)	IIB (n = 10)	IIC (n = 7)	*p*-value
Imaging features, n (%)^†^						
Empyema	20 (38.5)	1 (5.3)	9 (56.3)	6 (60.0)	4 (57.1)	**0.003**
Pulmonary infiltrates	27 (51.9)	5 (26.3)	12 (75.0)	7 (70.0)	3 (42.9)	**0.028**
Cavitary lesion	44 (84.6)	14 (73.7)	15 (93.8)	10 (100)	5 (71.4)	0.152
Symptom-to-Diagnosis Interval, days, median (IQR)	6 [5–9]	6 [5–9]	7 [5–10]	6 [5–10]	7 [5–11]	0.874
Surgical approach, n (%)^‡^						
Transcervical approach	40 (76.9)	18 (94.7)	11 (68.8)	4 (40.0)	7 (100.0)	** < 0.001**
Thoracic approach	12 (23.1)	1 (5.3)	5 (31.2)	6 (60.0)	0 (0.0)	
Postoperative stay, days, median (IQR)^‡§^	29 [21–37]	22 [20–33]	31 [24–40]	29 [23–37]	35 [33–41]	0.187
Reoperation, n (%)	7 (13.5)	2 (10.5)	0 (0.0)	2 (20.0)	3 (42.9)	0.108
90-day mortality, n (%)	6 (11.5)	2 (10.5)	2 (12.5)	2 (20.0)	0 (0.0)	0.772

^†^Patients may have multiple imaging features

^‡^Values presented as median (interquartile range); p-value from Kruskal–Wallis test

^§^Postoperative hospital stay analysis includes surviving patients only

*P*-values are from Fisher’s exact test for categorical variables unless otherwise specified. Significant *p*-values (< 0.05) are in bold

The median time from symptom onset to diagnosis was 6 days (IQR 5–9), with no significant differences across DNM types. The choice of surgical approach varied significantly with DNM type (*p* < 0.001). The transcervical approach was predominantly used for Type I and Type IIC infections (94.3% and 100%, respectively), while the thoracic approach was more frequent for Type IIB infections (60% vs. 40% for transcervical).

### Prognosis of DNM by staging

The median postoperative hospital stay was 28.5 days, with no significant differences among types (*p* = 0.187). Type I patients had the shortest median stay (22 days) and Type IIC the longest (35 days).

Seven patients required mediastinal reoperations. The reoperations rate was highest for Type IIC (42.9%) and zero for Type IIA, though the overall differences was not statistically significant (*p* = 0.108). The overall 90-day mortality was 11.5% (6/52), with two deaths each in Types I, IIA, and IIB. No mortality occurred in Type IIC.

Given the variation in surgical approach, we compared a composite outcome of reoperation or death within 90 days among patients with lower mediastinitis (Types IIA, IIB, IIC). The incidence was 33.3% in the transcervical group compared to 9.1% in the thoracic group (Table 3); this difference was not statistically significant (*p* = 0.227).

**Table 3:** Comparison of the 90-Day composite outcome (reoperation or death) by surgical approach in patients with lower mediastinitis (Types IIA, IIB, IIC)

Surgical approach	Total patients	Composite outcome, n (%)	*p*-value
Transcervical approach	21	7 (33.3%)	0.227
Thoracic approach	11	1 (9.1%)	

*P*-value was calculated using Fisher’s exact test

### Factors associated with clinical outcomes

We further investigated factors associated with patient prognosis, focusing on 90-day mortality (Table 4) and postoperative hospital stay (Table 5). Univariate logistic regression identified preoperative serum albumin as a significant predictor of 90-day mortality (OR 1.29 per 1 g/L decrease, 95% CI 1.02–1.63, *p* = 0.032). Similarly, a lower postoperative WBC count was significantly associated with increased mortality (OR 1.40 per 1 × 10^9^/L decrease, 95% CI 1.01–1.93, *p* = 0.046). Additionally, an odontogenic origin of infection was significantly associated with 90-day mortality (OR 0.122, 95% CI 0.019–0.776, *p* = 0.026). For postoperative hospital stay, a shorter symptom-to-diagnosis interval correlated with a longer hospitalization (Spearman’s r = − 0.386, *p* = 0.008). The presence of empyema was also associated with significantly prolonged hospitalization (median stay 34.5 days vs. 25.5 days, *p* = 0.020).

**Table 4:** Univariable logistic regression analysis of factors associated with 90-Day mortality

Predictor	Odds Ratio	95% CI	*p*-Value
Continuous, per 1-unit increase (+) or decrease (−)			
Age (year, +)	1.06	0.99–1.14	0.109
Preop. Albumin (g/L, −)	1.29	1.02–1.63	**0.032**
Preop. WBC (× 10^9^/L, −)	1.02	0.90–1.15	0.746
Preop. Neutrophils (%, +)	0.96	0.83–1.11	0.548
Postop. WBC (× 10^9^/L, −)	1.40	1.01–1.93	**0.046**
Postop. Albumin (g/L, −)	1.07	0.88–1.33	0.510
Symptom-to-Diagnosis (day, +)	1.03	0.89–1.18	0.735
Categorical			
Odontogenic Source (Yes vs. No)	0.12	0.02–0.78	**0.026**
Sex (Female vs. Male)	0.94	0.16–5.69	0.994
Smoker (Yes vs. No)	0.48	0.09–2.69	0.407
Diabetes (Yes vs. No)	1.17	0.19–7.09	0.862
Pulmonary Infiltrates (Yes vs. No)	0.18	0.02–1.69	0.135
Empyema (Yes vs. No)	1.29	0.21–7.76	0.784

*CI* confidence interval, *WBC* white blood cell count. Statistically significant results (*p* < 0.05) are in bold

**Table 5:** Factors associated with postoperative hospital stay

Predictor	Correlation / Group Comparison		*p*-value
Continuous	Spearman’s r	95% CI	
Age	− 0.24	− 0.50–0.07	0.111
Preop. Albumin (g/L)	0.12	− 0.19–0.40	0.440
Preop. WBC (× 10^9^/L)	− 0.16	− 0.44–0.14	0.275
Preop. Neutrophils (%)	− 0.25	− 0.51–0.06	0.099
Postop. WBC (× 10^9^/L)	− 0.18	− 0.46–0.12	0.227
Postop. Albumin (g/L)	0.05	− 0.25–0.35	0.722
Symptom-to-Diagnosis Interval	− 0.39	− 0.61–0.10	**0.008**

Categorical	Median Stay, Days (IQR)		
Odontogenic Source	Yes (n = 8)	22.0 (18.5–41.5)	0.116
	No (n = 44)	29.0 (22.0–36.0)	
Sex	Female (n = 18)	27.0 (21.5–41.5)	0.454
	Male (n = 34)	29.0 (22.0–35.0)	
Smoker	Yes (n = 18)	37.0 (28.5–41.5)	0.064
	No (n = 34)	26.5 (20.0–32.0)	
Diabetes	Yes (n = 19)	27.0 (20.0–41.0)	0.799
	No (n = 33)	29.0 (22.0–35.0)	
Pulmonary Infiltrates	Yes (n = 27)	31.5 (24.0–41.0)	0.149
	No (n = 25)	26.0 (20.0–32.0)	
Empyema	Yes (n = 20)	34.5 (27.5–41.5)	**0.020**
	No (n = 32)	25.5 (19.0–32.0)	

Analysis for continuous variables used Spearman’s rank correlation; for categorical variables, the independent-samples Mann–Whitney U test was used to compare median postoperative hospital stays. CI, confidence interval; IQR, interquartile range. Statistically significant results (*p* < 0.05) are in bold

## Discussion

DNM remains a formidable challenge in thoracic surgery, characterized by rapid progression and significant mortality [4, 13], as evidenced by the 11.5% 90-day mortality rate in our cohort. This analysis confirms the disease’s severity and suggests that prognostic factors extend beyond traditional anatomical classification to include a patient’s physiological status and infection aggressiveness.

In our study, the Endo classification guided the initial surgical approach but was not significantly associated with mortality and hospitalization, highlighting the limitations of a pure anatomical framework. The baseline characteristics and microbiological profile of our cohort were consistent with those reported in other centers [2, 8, 14, 15], reinforcing oropharyngeal and odontogenic origins as the primary sources [2, 3, 16]. Diabetes mellitus was the most prevalent comorbidity, which is also widely recognized as a potential risk factor for this condition [14]. However, in this study, it did not significantly increase mortality or prolong hospitalization. Notably, an odontogenic origin was a significant risk factor for mortality, warranting heightened vigilance.

CT imaging was crucial for diagnosing and staging [16, 17]. The differential distribution of empyema and pulmonary infiltrates across DNM types reflected the pathological progression, a pattern consistent with the general correlation between wider anatomical involvement and more severe imaging manifestations. Notably, among these features, only empyema emerged as the sole imaging feature significantly associated with extended hospitalization. The high prevalence of cavitary lesions justifies vigilant surveillance for aerodigestive tract perforation [18].

The absence of a universally surgical gold standard for DNM is due to a literature landscape dominated by small, single-center studies [7, 19, 20]. It is generally accepted that infections confined to the upper mediastinum can be drained via a transcervical approach, whereas involvement of the lower mediastinum requires drainage through a thoracic approach [5, 16, 21]. In our center, the transcervical approach was used for most upper mediastinal and Type IIC cases. This latter group demonstrated the highest reoperation rate and longest hospitalization, highlighting the challenge of adequate posterior mediastinal drainage via the neck.

For lower mediastinitis (Types IIA, IIB, IIC), we observed a clinical trend favoring the thoracic approach, with the composite outcome of reoperation or death being 33.3% in the transcervical group versus 9.1% in the thoracic group. Although this difference was not statistical significance—likely due to limited small sample size—it represents a clinically important trend consistent with the rationale that a more direct and extensive surgical exposure via thoracotomy or VATS may provide superior source control in the lower mediastinum [11, 12, 22]. This suggests that for Type IIB and other complex lower mediastinal infections, an initial thoracic approach should be strongly considered to avoid the high failure rate associated with transcervical drainage alone.

Based on the retrospective analysis of the seven cases requiring reoperation, the primary indications were the progression or development of new infectious foci (n = 4) and inadequate initial source control (n = 2), with one case attributed to drain occlusion. Notably, five of these seven patients had extensive lower or posterior mediastinal involvement (Type IIC or complex Type IIB) and were initially managed via a transcervical approach. This pattern underscores a key technical challenge: achieving definitive drainage in the lower and posterior mediastinum through a cervical incision alone can be insufficient for controlling aggressive, anatomically complex infections. These findings directly inform the clinical trend observed in our study, reinforcing the rationale for considering a thoracic approach as a potentially more definitive initial strategy for such advanced presentations to mitigate the risk of reoperation.

Furthermore, we identified key patient-specific prognostic factors. Although the difference in postoperative albumin levels did not reach statistical significance, a significantly lower preoperative albumin level was associated with 90-day mortality. This mandates the use of serum albumin as a key preoperative risk-stratification tool. Patients with significant hypoalbuminemia require aggressive nutritional support and should be recognized as having a substantially elevated mortality risk [23]. The association between a lower postoperative WBC count and increased mortality suggested that an inadequate immune response is a critical determinant of survival [24]. A shorter symptom-to-diagnosis interval was correlated with a longer hospitalization. This association suggests that a “hyperacute presentation” may reflect higher pathogen virulence or an exaggerated host inflammatory response, rather than being a mere marker of healthcare delay. The presence of empyema was another key driver of extended hospitalization.

Our study has several limitations. Its retrospective, single-center design may introduce selection and information biases. The small sample size, particularly in subgroup analyses, limited our statistical power and precluded multivariate analysis. Furthermore, the non-randomized selection of surgical approach may have influenced outcomes. To validate and generalize these findings, future prospective, multicenter registries are warranted, which could facilitate the development of risk-adjusted management algorithms to further personalize care for patients with DNM.

## Conclusion

Our experience confirms the utility of the Endo classification for stratifying DNM patients and guiding surgical management. We recommend a tailored approach: transcervical drainage for Types I, and a low threshold for a thoracic approach for Type IIB and other complex lower mediastinal infections. Critically, decision-making must integrate anatomical extent with patient physiology. Preoperative identification of high-risk patients—those with hypoalbuminemia, an odontogenic source, or a hyperacute presentation—should trigger a more aggressive multimodal strategy. The observed trend favoring the thoracic approach for lower mediastinitis warrants validation in larger, multi-center studies.

## Data Availability

The data underlying this article will be shared on reasonable request to the corresponding author.
